# The effectiveness of non-pharmacological treatments for auditory verbal hallucinations in schizophrenia spectrum disorders: a systematic review and meta-analysis

**DOI:** 10.1192/j.eurpsy.2026.10537

**Published:** 2026-07-31

**Authors:** M. Cobandag, N. Sigala

**Affiliations:** 1 Department of Clinical Neuroscience; 2 Departmenr of Clinical Neuroscience, Brighton and Sussex Medical School, Brighton, United Kingdom

## Abstract

**Introduction:**

Schizophrenia is a chronic severe mental illness affecting 24-million people globally, associated with a life expectancy 15 years shorter than the general population. Approximately 70% of people with schizophrenia experience auditory verbal hallucinations (AVHs), i.e. ‘hearing voices’. Current treatment approaches remain unsuccessful at treating AVHs in up to 30% of cases.

**Objectives:**

This systematic review and meta-analysis evaluated randomised controlled trials (RCTs) of potential non-pharmacological treatments for AVHs in schizophrenia spectrum disorders. The effectiveness of emerging treatments was assessed and gaps in research were identified, with implications for clinical practice.

**Methods:**

A literature search was performed between 2013-2024 across five databases: *PubMed, Embase, PsycINFO, Medline,* and *Web of Science.* The meta-analysis included 45 studies, based on predefined criteria and an assessment for bias. Effect sizes (Hedge’s g) were calculated for each study and overall, using a random effects model with 95% confidence intervals. The study was conducted in accordance with *PRISMA* guidelines and was pre-registered (PROSPERO ID: CRD42024598615).

**Results:**

Our sample included 2,314 patients and fourteen different interventions. The calculated overall mean effect size for all interventions was -0.298 (95% CI, [-0.470,-0.126]), representing a medium size and statistically significant effect. Subgroup analyses revealed that both AVATAR therapy and cognitive behavioural therapy (CBT) subgroups had medium size and statistically significant effects. Conversely, repetitive transcranial magnetic stimulation (rTMS) and transcranial direct current stimulation (tDCS) showed small size and not statistically significant effects.

**Conclusions:**

AVATAR therapy has the strongest evidence for treating AVHs, highlighting the need for large-scale RCTs and consideration for integration into treatment guidelines. CBT requires standardisation in methodology for reliability, and clinical guidelines should focus on symptomology rather than diagnosis. Acceptance and commitment therapy shows promise but requires further high-quality RCTs. Non-invasive brain stimulation techniques require further trials before they can be considered for clinical use.

**Disclosure of Interest:**

None Declared

